# Application of multiple machine learning approaches to determine key pyroptosis molecules in type 2 diabetes mellitus

**DOI:** 10.3389/fendo.2023.1112507

**Published:** 2023-07-19

**Authors:** Min Wang, He Wu, Ronghua Wu, Yongshun Tan, Qingqing Chang

**Affiliations:** ^1^ Department of Clinical Laboratory, The Affiliated People’s Hospital of Shandong First Medical University, Jinan, China; ^2^ Department of Endocrinology, The Affiliated People’s Hospital of Shandong First Medical University, Jinan, China; ^3^ Department of Endocrinology, The Third People’s Hospital of Jinan, Jinan, China; ^4^ Department of Nephrology, The Affiliated People’s Hospital of Shandong First Medical University, Jinan, China

**Keywords:** type 2 diabetes mellitus, pyroptosis genes, machine learning, support vector machine, immune infiltration, druggable targets, risk

## Abstract

**Objective:**

Pyroptosis, a lytic and inflammatory programmed cell death, has been implicated in type 2 diabetes mellitus (T2DM) and its complications. Nonetheless, it remains elusive exactly which pyroptosis molecule exerts an essential role in T2DM, and this study aims to solve such issue.

**Methods:**

Transcriptional profiling datasets of T2DM, i.e., GSE20966, GSE95849, and GSE26168, were acquired. Four machine learning models, namely, random forest, support vector machine, extreme gradient boosting, and generalized linear modeling, were built based on pyroptosis genes. A nomogram of key pyroptosis genes was also generated, and the clinical value was appraised *via* calibration curves and decision curve analysis. Immune infiltration was inferred utilizing CIBERSORT. Drug–druggable target relationships were acquired from the Drug Gene Interaction Database. Through WGCNA, key pyroptosis-relevant genes were selected.

**Results:**

Most pyroptosis genes exhibited upregulation in T2DM relative to controls, indicating the activity of pyroptosis in T2DM. The SVM model composed of BAK1, CHMP2B, NLRP6, PLCG1, and TIRAP exhibited the best performance in T2DM diagnosis, with AUC = 1. The nomogram can predict the risk of T2DM for clinical practice. NK cells resting exhibited a lower abundance in T2DM *versus* normal specimens, with a higher abundance of neutrophils. NLRP6 was positively linked with neutrophils. Drugs (keracyanin, 9,10-phenanthrenequinone, diclofenac, phosphomethylphosphonic acid adenosyl ester, acetaminophen, cefixime, aspirin, ustekinumab) potentially targeted the key pyroptosis genes. Additionally, CHMP2B-relevant genes were determined.

**Conclusion:**

Altogether, this work proposes the key pyroptosis genes in T2DM, which might become possible molecules for the management and treatment of T2DM and its complications.

## Introduction

Diabetes mellitus (DM) is a chronic metabolic disorder with the characteristics of high blood glucose level, which results from definitive insulin function and/or decreased insulin generation (1). In both type 1 DM (T1DM) and type 2 DM (T2DM), a variety of genetic and environmental factors may lead to progressive loss of β-cell number and/or function, clinically manifested as hyperglycemia (2–4). When hyperglycemia occurs, diabetic individuals are at risk of developing the same chronic complications, though the rate of progression may be different (5). DM is connected with acute and chronic complications, which can be restrained or delayed through intensive glycemic management (6). An in-depth understanding of the pathogenesis of T2DM allows us to better predict the outcome and choose the more precise treatment.

Pyroptosis is a form of programmed cell death characterized by rapid membrane rupture, cell swelling with large bubbles, and the release of proinflammatory cell ingredients (7). The main role of pyroptosis is to drive a strong inflammatory response and protect the host from microbial infection (8). Accumulated evidence suggests the connections of pyroptosis with DM and its complications. For instance, CD74 ablation can rescue T2DM-driven cardiac remodeling and contractile dysfunction *via* pyroptosis-induced modulation of ferroptosis (9). HECTD3 facilitates NLRP3 inflammasome and pyroptosis for exacerbating DM-relevant cognitive impairment through stabilizing MALT1 (10). Mitochondrial injury and activation of the cytosolic DNA sensor cGAS-STING signaling result in cardiac pyroptosis and hypertrophy in diabetic cardiomyopathy (11). ManNAc exerts a protective effect on podocyte pyroptosis in diabetic renal injury through inhibition of mitochondrial injury and ROS/NLRP3 signaling (12). Schisandrin A mitigates ferroptosis and NLRP3 inflammasome-driven pyroptosis in diabetic nephropathy *via* mitochondrial damage through AdipoR1 ubiquitination (13). Nonetheless, it is still elusive exactly which pyroptosis molecule exerts a crucial function in T2DM pathogenesis. Herein, diverse machine learning algorithms were adopted for the selection of key pyroptosis molecules, which might have the potential as therapeutic targets of T2DM.

## Materials and methods

### Datasets

Transcriptional profiling of DM was acquired from the Gene Expression Omnibus. The GSE20966 dataset (https://www.ncbi.nlm.nih.gov/geo/query/acc.cgi?acc=GSE20966) was composed of pancreatic tissues from 10 non-diabetic controls and 10 T2DM patients on the GPL1352 platform (14). The GSE95849 dataset (https://www.ncbi.nlm.nih.gov/geo/query/acc.cgi?acc=GSE95849) comprised peripheral blood samples from six T2DM patients and six healthy participants on the GPL22448 platform (15). The GSE20966 and GSE95849 datasets were merged as the discovery set, and batch effects were removed using the sva package, which was visualized into the principal component analysis (PCA) (16). The GSE26168 dataset (https://www.ncbi.nlm.nih.gov/geo/query/acc.cgi?acc=GSE26168) contained peripheral blood specimens from eight healthy subjects and nine T2DM patients on the GPL6883 platform for external verification (17).

### Collection of pyroptosis genes

Pyroptosis genes were gathered from prior research (18–20). RCircos package was adopted for the visualization of the genomic position of pyroptosis genes (21).

### Machine learning models

Four machine learning approaches composed of random forest (RF), support vector machine (SVM), extreme gradient boosting (XGB), and generalized linear modeling (GLM) were conducted for selecting the characteristic pyroptosis genes. Receiver operating characteristic curves (ROCs) were plotted for computing the area under the curve (AUC) on each established model or key pyroptosis gene.

### Nomogram establishment

A nomogram was generated through the integration of key pyroptosis genes utilizing the rms package. Calibration curves were drawn for the visualization and evaluation of the consistency between the actual observations and the nomogram-predicted results. Decision curve analysis (DCA) was conducted for quantifying the net benefit at distinct threshold probabilities.

### Gene set enrichment analysis

Gene set enrichment analysis (GSEA) was conducted for identifying the possible functions of the selected genes (22). The reference gene sets were acquired from the Molecular Signature Database (23), with *p <*0.05 as the threshold.

### Immune infiltration estimation

Through the CIBERSORT approach (24), the normalized transcriptional profiling of T2DM and normal specimens was transformed to the immune components based upon the LM22 reference signature matrix set at 1,000 permutations.

### Drug–druggable target network

From the Drug Gene Interaction Database (DGIdb; www.dgidb.org) (25), the interactions of drugs with key pyroptosis genes were acquired. Afterward, a drug–target network was built by using the Cytoscape software (26).

### Weighted correlation network analysis

The weighted correlation network analysis (WGCNA) package was adopted for building co-expression modules (27). The optimal soft thresholding value was selected through the pickSoftThreshold function. By using the dynamic tree cut method, highly connected genes were merged into one co-expression module. The structure of the co-expression modules was visualized through a heatmap plot *via* the TOMplot function. The interactions of modules with key pyroptosis genes were then estimated *via* Pearson’s test, followed by the evaluation of the module membership *versus* gene significance.

### Protein–protein interaction

Module genes were imported onto the STRING website (28), and protein–protein interaction pairs were acquired. The key genes were selected by using molecular complex detection (MCODE) (a plugin in Cytoscape).

### Functional enrichment analysis

By using the clusterProfiler method (29), Gene Ontology (GO) enrichment analysis was carried out, which comprised the biological process (BP), cellular component (CC), and molecular function (MF). Afterward, enrichment of the Kyoto Encyclopedia of Genes and Genomes (KEGG) pathways was implemented. Results with *p <*0.05 were indicative of significant enrichment.

### Statistical analysis

All the analyses were implemented using the R package (version 3.5.3; https://www.r-project.org/). Two groups were compared by adopting the Wilcoxon test. Pearson’s test was employed for correlation analysis. *p <*0.05 was indicative of statistical significance.

## Results

### Aberrant expression of pyroptosis genes in T2DM

**Figure 1:**
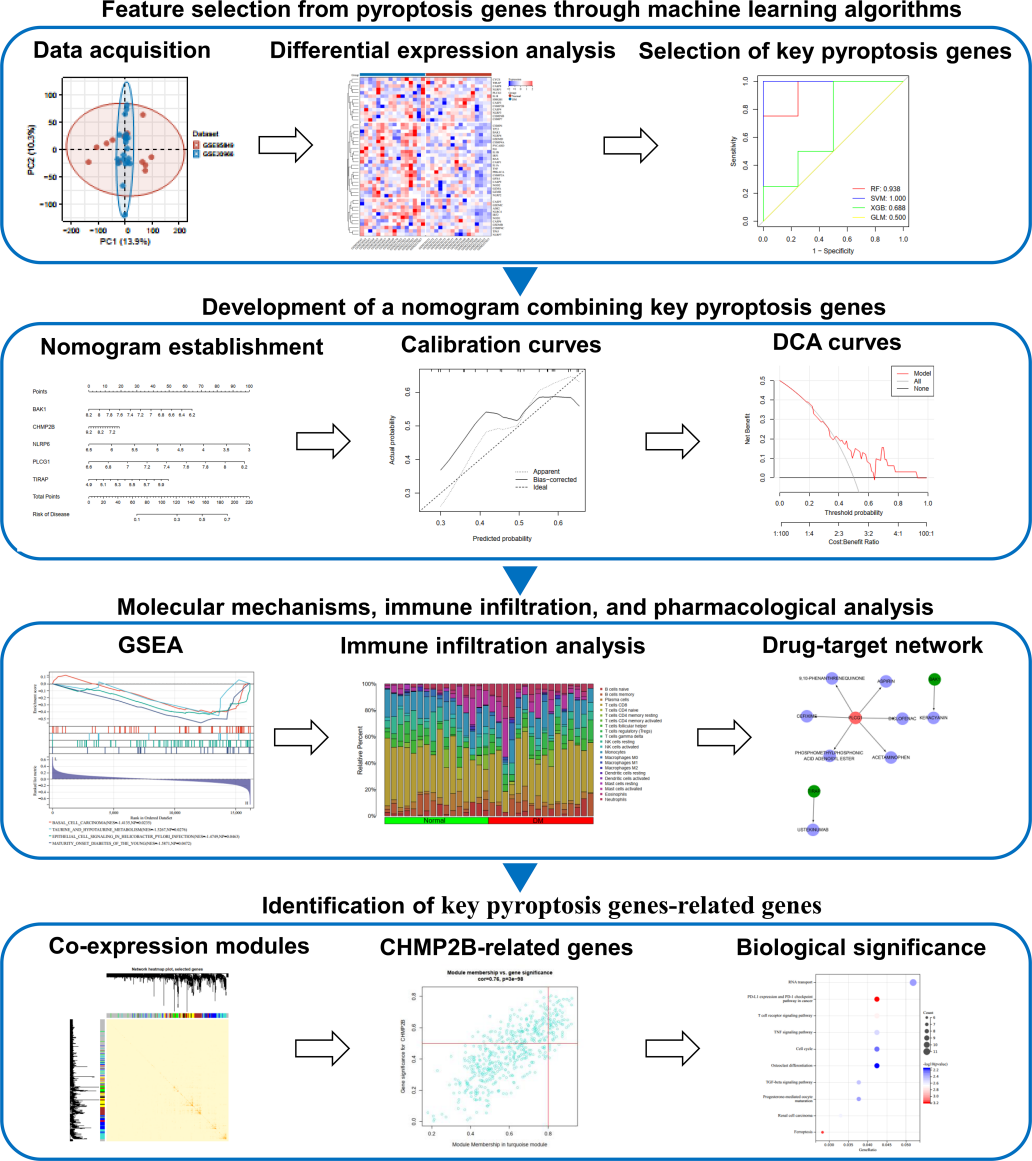
The workflow of this study.

**Figure 2:**
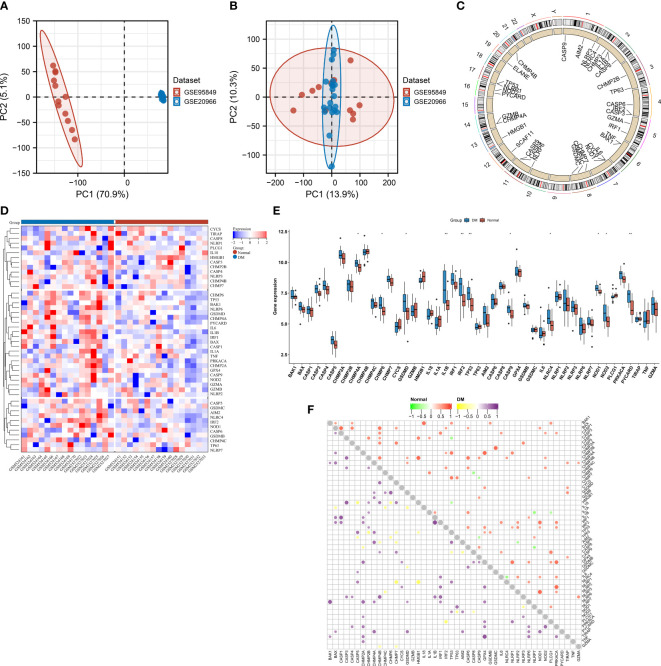
Aberrant expression of pyroptosis genes in type 2 diabetes mellitus (T2DM). **(A, B)** PCA plots of transcriptional profiling of T2DM and control specimens in the GSE20966 and GSE95849 datasets **(A)** before and **(B)** after the removal of batch effects. Each dot denotes one specimen. **(C)** Genomic location of pyroptosis genes. **(D)** Heatmap of the transcript levels of pyroptosis genes across T2DM and controls. **(E)** Comparing the levels of pyroptosis genes in T2DM relative to normal specimens. *p < 0.05; **p < 0.01. **(F)** Relationships between pyroptosis genes in T2DM or normal tissues.

**Figure 1** depicts the workflow of this study. This work combined two T2DM datasets, namely, GSE20966 and GSE95849, for expanding the sample size as much as possible (**Figure 2A**). The removal of batch effects was then implemented (**Figure 2B**). **Figure 2C** illustrates the genomic position of pyroptosis genes. The detailed information is listed in **Table 1**. Next, the expression differences in pyroptosis genes were estimated in T2DM and controls. Most pyroptosis genes including CHMP4A, CHMP6, GSDMD, IL1B, IRF2, TP53, CASP9, NLRC4, NOD1, NOD2, and PYCARD exhibited notable upregulation in T2DM relative to normal specimens (**Figures 2D, E**), indicating the activation of pyroptosis in T2DM. Both in T2DM and control tissues, pyroptosis genes closely interacted (**Figure 2F**).

**Table 1:** Information on the genomic position of pyroptosis genes.

Gene	Chromosome	Start	End
*CASP9*	chr1	15490832	15526534
*AIM2*	chr1	159062484	159147096
*NLRP3*	chr1	247416156	247449108
*NLRC4*	chr2	32224453	32265854
*CHMP3*	chr2	86503431	86563479
*IL1A*	chr2	112773915	112784590
*IL1B*	chr2	112829751	112836903
*CASP8*	chr2	201233443	201287711
*CHMP2B*	chr3	87227271	87255548
*TP63*	chr3	189631416	189897279
*CASP6*	chr4	109688622	109703583
*IRF2*	chr4	184387713	184474580
*CASP3*	chr4	184627696	184649509
*GZMA*	chr5	55102648	55110252
*IRF1*	chr5	132481609	132490798
*TNF*	chr6	31575567	31578336
*BAK1*	chr6	33572547	33580293
*IL6*	chr7	22725884	22732002
*CYCS*	chr7	25120091	25125361
*NOD1*	chr7	30424527	30478784
*CHMP7*	chr8	23243637	23262000
*CHMP4C*	chr8	81732434	81759515
*GSDMC*	chr8	129748196	129786888
*GSDMD*	chr8	143553207	143563062
*NLRP6*	chr11	278365	285359
*CASP4*	chr11	104942866	104969436
*CASP5*	chr11	104994235	105023168
*CASP1*	chr11	105025443	105035250
*IL18*	chr11	112143251	112164117
*TIRAP*	chr11	126283065	126298845
*SCAF11*	chr12	45919131	45992120
*HMGB1*	chr13	30456704	30617597
*CHMP4A*	chr14	24209583	24213869
*GZMB*	chr14	24630954	24634267
*PYCARD*	chr16	31201485	31203450
*NOD2*	chr16	50693603	50733077
*NLRP1*	chr17	5499427	5619424
*TP53*	chr17	7661779	7687550
*GSDMB*	chr17	39904595	39919854
*GSDMA*	chr17	39962973	39977766
*CHMP6*	chr17	80991598	81009517
*CHMP4B*	chr20	33811304	33854366
*PLCG1*	chr20	41136960	41196801
*ELANE*	chr19	851014	856247
*GPX4*	chr19	1103926	1106791
*PRKACA*	chr19	14091688	14118084
*BAX*	chr19	48954815	48961798
*NLRP7*	chr19	54923509	54966312
*NLRP2*	chr19	54953130	55001142
*CHMP2A*	chr19	58551566	58555124

### Establishment of multiple machine learning models of T2DM based upon pyroptosis genes

**Figure 3:**
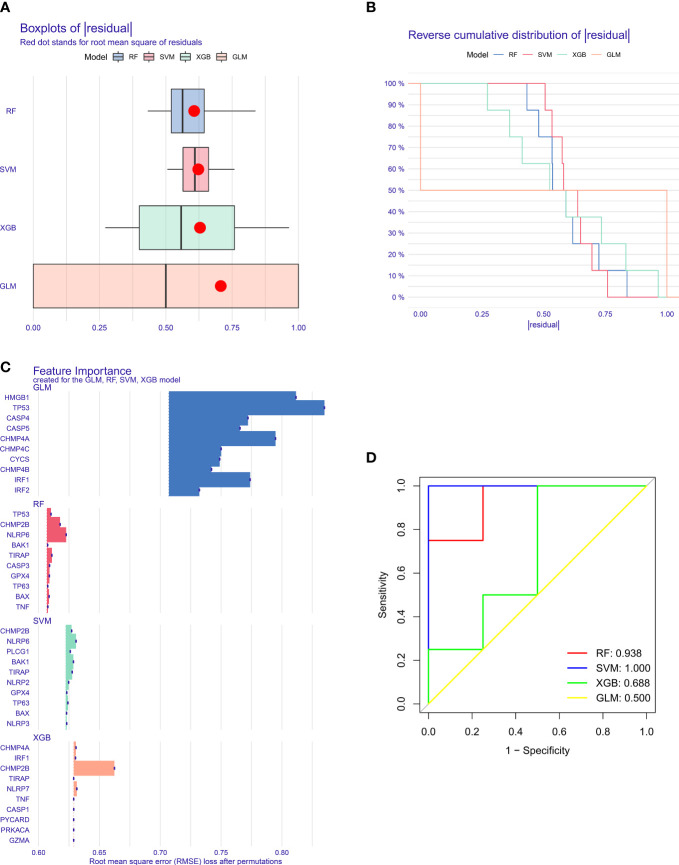
Selection of key pyroptosis genes through multiple machine learning approaches. **(A)** Box plots showing the “residual” of each machine learning method. **(B)** Reverse cumulative distribution of the “residual” for diverse machine learning approaches. **(C)** Feature importance of the selected pyroptosis genes by different machine learning approaches. **(D)** ROCs for the assessment of the diagnostic efficacy of distinct machine learning models.

Four machine learning algorithms—RF, SVM, XGB, and GLM—were implemented for establishing pyroptosis-relevant models for T2DM diagnosis. The selected characteristic pyroptosis genes of each model were as follows: RF (CASP3, TP53, TIRAP, CHMP2B, and NLRP6), SVM (PLCG1, CHMP2B, TIRAP, BAK1, and NLRP6), XGB (CASP1, IRF1, CHMP4A, NLRP7, and CHMP2B), and GLM (CASP4, IRF1, CHMP4A, HMGB1, and TP53). Among the four models, the SVM model exhibited the lowest “residual” (**Figures 3A, B**). We also summarized the feature importance distribution of pyroptosis genes in each machine learning algorithm (**Figure 3C**). The ROCs demonstrated excellent diagnostic efficacy of the SVM model with AUC = 1 (**Figure 3D**). The sensitivity of the RF, SVM, XGB, and GLM models was 1, 1, 1, and 0.75, respectively. The specificity of the four models was 0.75, 1, 0.5, and 0.25, respectively. Thus, the SVM was the best model, and the genes selected by the SVM were considered the key pyroptosis genes.

### Generation of a nomogram based upon key pyroptosis genes for T2DM risk

**Figure 4:**
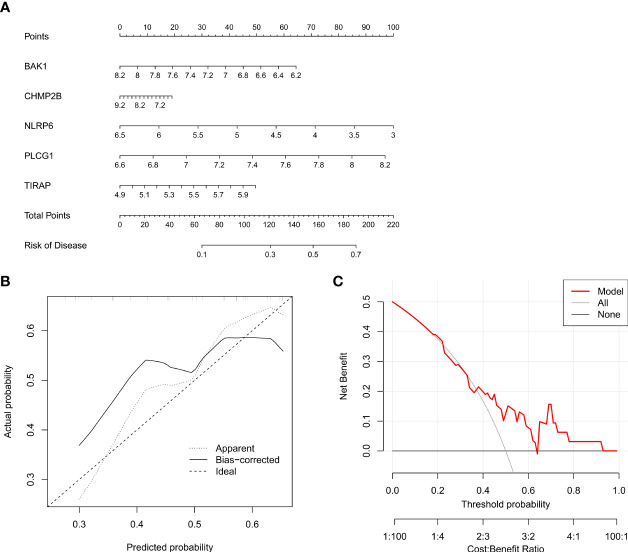
Generation of a nomogram for T2DM based upon key pyroptosis genes. **(A)** The nomogram comprising BAK1, CHMP2B, NLRP6, PLCG1, and TIRAP. **(B)** Calibration curves showing the consistency between the nomogram-predicted results and actual observations. **(C)** DCA curves for the evaluation of the clinical net benefit.

Five key pyroptosis genes were eventually selected for establishing the nomogram, composed of BAK1, CHMP2B, NLRP6, PLCG1, and TIRAP (**Figure 4A**). Calibration curves proved the good consistency between the nomogram-predictive results and the actual observations (**Figure 4B**). For determining the clinical significance of the nomogram in daily clinical practice, we plotted DCA curves. As depicted in **Figure 4C**, in comparison to all of the patients or none of them, the application of the nomogram to predict the risk of T2DM might be reasonable and have more clinical net benefit in accordance with the predicted possibilities computed by the nomogram and threshold probabilities.

### Verification of the diagnostic efficacy of the SVM model

**Figure 5:**
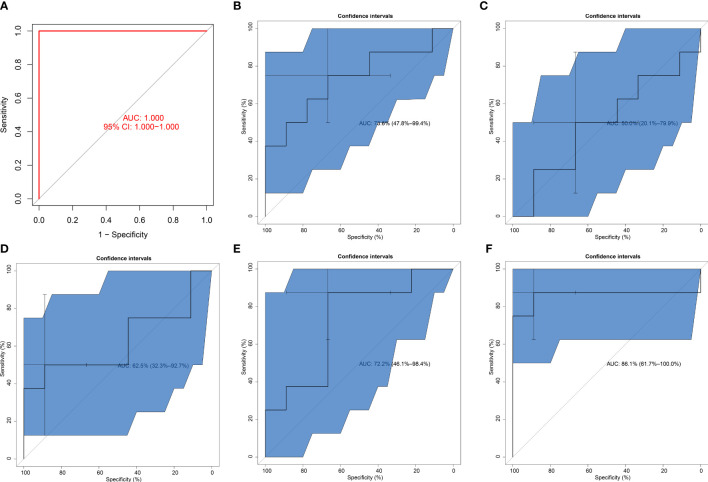
Verification of the diagnostic efficacy of the SVM model. **(A)** External verification of the diagnostic performance of the SVM model in the GSE26168 dataset. **(B–F)** ROCs of **(B)** BAK1, **(C)** CHMP2B, **(D)** NLRP6, **(E)** PLCG1, and **(F)** TIRAP in diagnosing T2DM.

The excellent diagnostic efficacy of the SVM model was also proven in the GSE26168 dataset (AUC = 1; **Figure 5A**). In addition, this work investigated the diagnostic performance of each key pyroptosis gene. It was demonstrated that BAK1, CHMP2B, NLRP6, PLCG1, and TIRAP can individually diagnose T2DM with relatively high AUC values (**Figures 5B–F**). This demonstrated the crucial significance of key pyroptosis genes in T2DM.

### Molecular mechanisms underlying key pyroptosis genes and their correlations with clinical features

**Figure 6:**
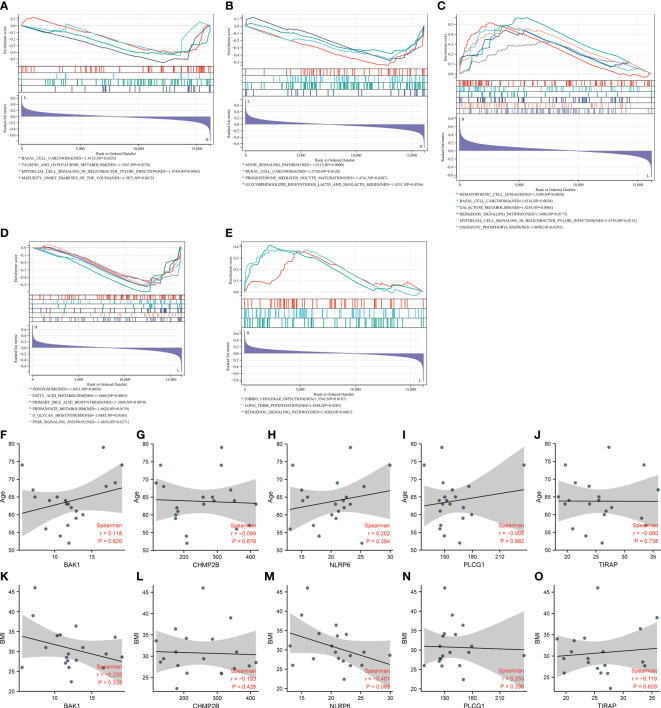
Molecular mechanisms underlying key pyroptosis genes. **(A–E)** GSEA for the difference in enriched KEGG pathways between low and high expression of **(A)** BAK1, **(B)** CHMP2B, **(C)** NLRP6, **(D)** PLCG1, and **(E)** TIRAP. **(F–J)** Correlation analysis on **(F)** BAK1, **(G)** CHMP2B, **(H)** NLRP6, **(I)** PLCG1, and **(J)** TIRAP with age. **(K–O)** Correlation analysis on **(K)** BAK1, **(L)** CHMP2B, **(M)** NLRP6, **(N)** PLCG1, and **(O)** TIRAP with BMI.

GSEA unveiled that BAK1 was negatively connected with basal cell carcinoma, taurine and hypotaurine metabolism, epithelial cell signaling in *Helicobacter pylori* infection, and maturity-onset diabetes of the young (**Figure 6A**). CHMP2B was negatively related to the mTOR signaling pathway, renal cell carcinoma, progesterone-mediated oocyte maturation, and glycosphingolipid biosynthesis of lacto- and neolacto-series (**Figure 6B**). NLRP6 exhibited positive interactions with hematopoietic cell lineage, basal cell carcinoma, galactose metabolism, hedgehog signaling pathway, epithelial cell signaling in *H. pylori* infection, and oxidative phosphorylation (**Figure 6C**). PLCG1 was negatively linked with peroxisome, fatty acid metabolism, primary bile acid biosynthesis, propanoate metabolism, O-glycan biosynthesis, and PPAR signaling pathway (**Figure 6D**). TIRAP presented positive connections with *Vibrio cholerae* infection, long-term potentiation, and hedgehog signaling pathway (**Figure 6E**).

We also evaluated the correlations between the key pyroptosis genes and clinical features (age and BMI). Nevertheless, no significant associations between BAK1, CHMP2B, NLRP6, PLCG1, and TIRAP and age and BMI were observed among T2DM patients (**Figures 6F–O**).

### Interactions of key pyroptosis genes with immune infiltration

**Figure 7:**
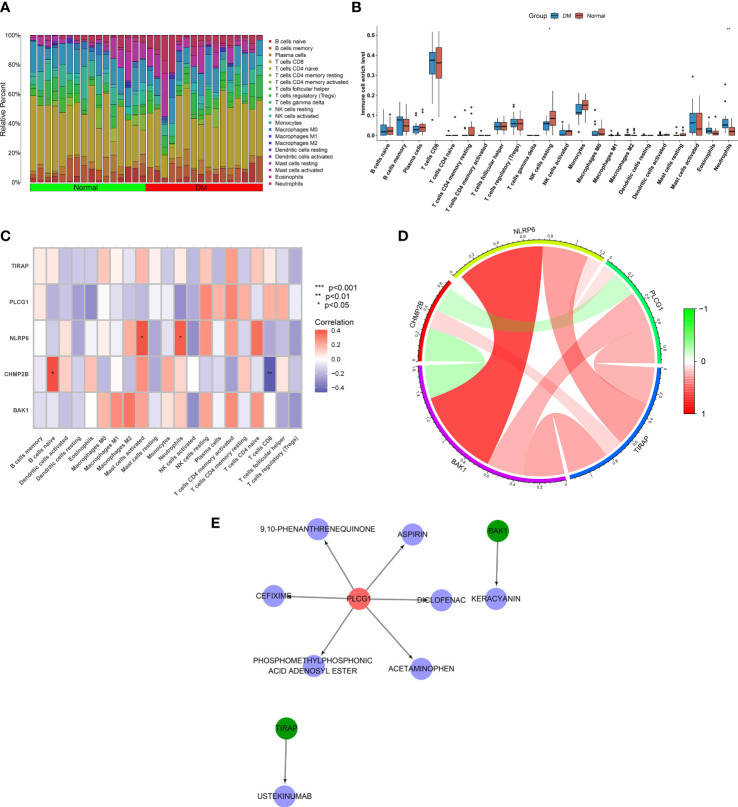
Associations of key pyroptosis genes with immune infiltration and generation of a drug–druggable target network. **(A)** Landscape of the fraction of immune components across T2DM and normal specimens. **(B)** Comparing the fraction of immune components in T2DM relative to controls. **(C)** Correlation analysis on key pyroptosis genes with immune infiltration. **(D)** Interactions between key pyroptosis genes. **(E)** A network of key pyroptosis genes with matched compounds. *p < 0.05; **p < 0.01.

Through the implementation of CIBERSORT, the fraction of diverse immune cell types was estimated across T2DM and control tissues (**Figure 7A**; **Supplementary Table 1**). Their difference was also investigated between the two groups. As illustrated in **Figure 7B**, natural killer (NK) cells resting exhibited lower abundance in T2DM relative to the normal specimens. In contrast, the higher abundance of neutrophils was investigated in T2DM. Among the key pyroptosis genes, NLRP6 was positively connected with mast cells activated and neutrophils (**Figure 7C**). In addition, CHMP2B had a positive interaction with B cells naive. The assessment of the interactions between key pyroptosis genes was also conducted. In **Figure 7D**, CHMP2B negatively interacted with BAK1 and PLCG1, while the other genes presented positive interactions.

### Drug–druggable target network

Drugs that potentially targeted key pyroptosis genes were inferred utilizing the DGIdb. As a result, BAK1 was a druggable target of keracyanin; PLCG1 was a druggable target of 9,10-phenanthrenequinone, diclofenac, phosphomethylphosphonic acid adenosyl ester, acetaminophen, cefixime, and aspirin; and TIRAP was a druggable target of ustekinumab (**Figure 7E**).

### Establishment of the key pyroptosis gene-relevant co-expression modules

**Figure 8:**
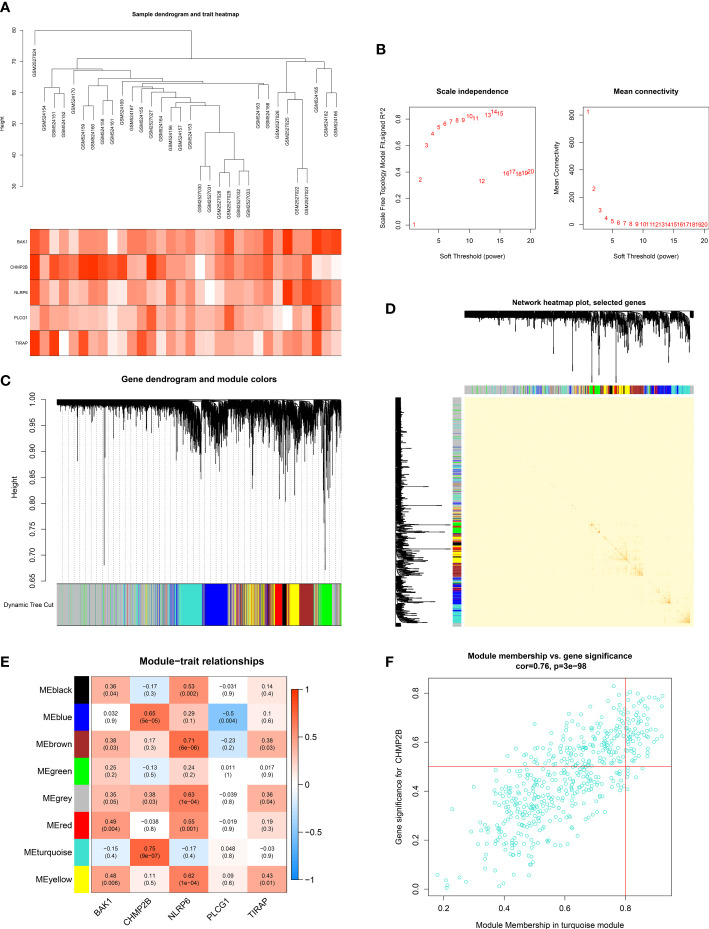
Establishment of key pyroptosis gene-relevant co-expression modules. **(A)** Sample dendrogram (upper) and heatmap of key pyroptosis genes (below). **(B)** Selection of the appropriate soft thresholding value in accordance with scale independence as well as mean connectivity. **(C)** Gene dendrogram and merged modules. **(D)** The network heatmap plot. **(E)** Pearson correlation on the established modules with key pyroptosis genes. Red, positive interaction; blue, negative interaction. **(F)** Scatter plot of the module membership in the turquoise module *versus* gene significance for CHMP2B.

To select the key pyroptosis gene-relevant co-expression modules, this work adopted the WGCNA (**Figure 8A**). The appropriate soft thresholding value was set to 13 based on the scale independence as well as mean connectivity (**Figure 8B**). By using the dynamic tree cut method, eight co-expression modules were built (**Figures 8C, D** **)**. Among them, the turquoise module exhibited the strongest interaction with the key pyroptosis gene CHMP2B (**Figure 8E**). The genes in the turquoise module were regarded as CHMP2B-relevant genes (**Figure 8F**; **Supplementary Table 2**).

### Interactions between key CHMP2B-relevant genes and their biological implications

**Figure 9:**
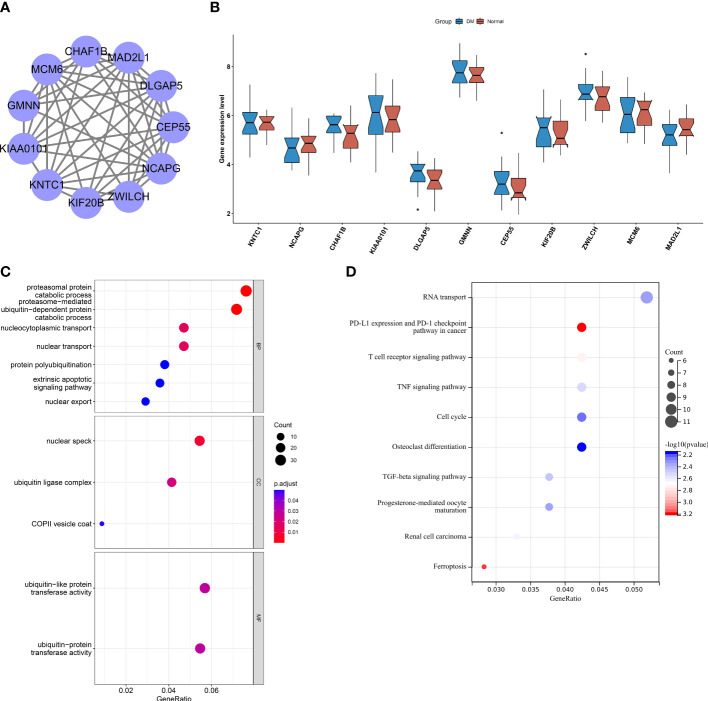
Interactions between key CHMP2B-relevant genes and their biological implications. **(A)** Protein interactions between key CHMP2B-relevant genes. **(B)** Transcript level of key CHMP2B-relevant genes in T2DM and control tissues. **(C)** GO enrichment results of key CHMP2B-relevant genes. **(D)** KEGG pathways enriched by CHMP2B-relevant genes.

To determine the key CHMP2B-relevant genes, the MCODE method was adopted (**Supplementary Table 3**). As a result, 10 key genes were acquired as follows: KNTC1, NCAPG, KIAA0101, DLGAP5, GMNN, CEP55, KIF20B, ZWILCH, MCM6, and MAD2L1 (**Figure 9A**). Most of the genes presented differential expression in T2DM relative to the normal specimens (**Figure 9B**). The biological significance of CHMP2B-relevant genes was further probed. It was noted that they were notably connected with proteasome-mediated ubiquitin-dependent protein catabolic process, proteasomal protein catabolic process, etc. (**Figure 9C**; **Table 2**). In addition, RNA transport, cell cycle, T-cell receptor pathway, etc. were remarkably enriched by CHMP2B-relevant genes (**Figure 9D**; **Table 3**).

**Table 2:** GO enrichment results of CHMP2B-relevant genes.

GO	ID	Description	Gene ratio	*p*-value	Count
BP	GO:0043161	Proteasome-mediated ubiquitin-dependent protein catabolic process	32/446	5.08E−09	32
BP	GO:0010498	proteasomal protein catabolic process	34/446	3.36E−08	34
BP	GO:0006913	Nucleocytoplasmic transport	21/446	1.60E−05	21
BP	GO:0051169	Nuclear transport	21/446	1.60E−05	21
BP	GO:0000209	Protein polyubiquitination	17/446	7.80E−05	17
BP	GO:0097191	Extrinsic apoptotic signaling pathway	16/446	8.03E−05	16
BP	GO:0051168	Nuclear export	13/446	9.11E−05	13
CC	GO:0016607	Nuclear speck	25/459	1.49E−05	25
CC	GO:0000151	Ubiquitin ligase complex	19/459	0.000101	19
CC	GO:0030127	COPII vesicle coat	4/459	0.00033	4
MF	GO:0019787	Ubiquitin-like protein transferase activity	26/457	8.48E−05	26
MF	GO:0004842	Ubiquitin-protein transferase activity	25/457	8.86E−05	25

**Table 3:** KEGG pathways enriched by CHMP2B-relevant genes.

ID	Description	Gene ratio	*p*-value	Count
hsa05235	PD-L1 expression and PD-1 checkpoint pathway in cancer	9/212	0.000595	9
hsa04216	Ferroptosis	6/212	0.000617	6
hsa04660	T-cell receptor signaling pathway	9/212	0.00182	9
hsa05211	Renal cell carcinoma	7/212	0.002366	7
hsa04668	TNF signaling pathway	9/212	0.003029	9
hsa04350	TGF-beta signaling pathway	8/212	0.00359	8
hsa04914	Progesterone-mediated oocyte maturation	8/212	0.004634	8
hsa03013	RNA transport	11/212	0.004697	11
hsa04110	Cell cycle	9/212	0.005942	9
hsa04380	Osteoclast differentiation	9/212	0.007286	9
hsa05321	Inflammatory bowel disease (IBD)	6/212	0.007635	6
hsa05210	Colorectal cancer	7/212	0.008047	7
hsa04068	FoxO signaling pathway	9/212	0.008437	9
hsa05221	Acute myeloid leukemia	6/212	0.008828	6
hsa05230	Central carbon metabolism in cancer	6/212	0.010148	6
hsa04120	Ubiquitin-mediated proteolysis	9/212	0.010658	9
hsa04210	Apoptosis	9/212	0.010658	9
hsa04140	Autophagy—animal	9/212	0.01115	9
hsa04630	Jak–STAT signaling pathway	10/212	0.011585	10
hsa04550	Signaling pathways regulating the pluripotency of stem cells	9/212	0.01273	9
hsa04722	Neurotrophin signaling pathway	8/212	0.014365	8
hsa05215	Prostate cancer	7/212	0.015054	7
hsa05164	Influenza A	10/212	0.01583	10
hsa05133	Pertussis	6/212	0.015876	6
hsa05220	Chronic myeloid leukemia	6/212	0.015876	6
hsa05213	Endometrial cancer	5/212	0.019095	5
hsa04620	Toll-like receptor signaling pathway	7/212	0.021311	7
hsa04066	HIF-1 signaling pathway	7/212	0.026759	7
hsa04010	MAPK signaling pathway	14/212	0.026926	14
hsa04940	Type I diabetes mellitus	4/212	0.027342	4
hsa04929	GnRH secretion New!	5/212	0.027964	5
hsa04137	Mitophagy—animal	5/212	0.029658	5
hsa05162	Measles	8/212	0.031727	8
hsa05166	Human T-cell leukemia virus 1 infection	11/212	0.03316	11
hsa04141	Protein processing in endoplasmic reticulum	9/212	0.034106	9
hsa04973	Carbohydrate digestion and absorption	4/212	0.036408	4
hsa04657	IL-17 signaling pathway	6/212	0.038241	6
hsa05135	Yersinia infection	7/212	0.041916	7
hsa05200	Pathways in cancer	21/212	0.045382	21
hsa01524	Platinum drug resistance	5/212	0.045537	5
hsa04710	Circadian rhythm	3/212	0.049148	3

## Discussion

Pyroptosis, a proinflammatory form of programmed cell death, has the features of cellular swelling, lysis, and the release of proinflammatory cytokines (30). In the present work, most pyroptosis molecules containing CHMP4A, CHMP6, GSDMD, IL1B, IRF2, TP53, CASP9, NLRC4, NOD1, NOD2, and PYCARD presented remarkable upregulation in T2DM relative to controls, which was indicative of the activation of the pyroptosis process in T2DM, similar to prior research (31).

To select the key pyroptosis molecules exerting essential functions in T2DM, four machine learning algorithms—RF, SVM, XGB, and GLM—were applied. Among them, the SVM model presented the best efficacy in T2DM prediction. Therefore, SVM-selected genes were considered key pyroptosis molecules, including BAK1, CHMP2B, NLRP6, PLCG1, and TIRAP. The nomogram built had clinical superiority in risk prediction. Experimental research has demonstrated that the key pyroptosis genes are involved in DM. Targeting BAK1 alleviates diabetic cardiomyopathy (32). In addition, inhibition of PLCG1 mitigates diabetic retinopathy (33). TIRAP is associated with T2DM and insulin resistance (34).

T2DM is associated with increased systemic inflammation that results in insulin resistance, hyperglycemia, and risk of diabetic complications (35). Herein, it was found that T2DM displayed a lower level of NK cells resting as well as a higher level of neutrophils in comparison to normal specimens, consistent with prior research (36). It has been proven that NK cells correlate to DM by relieving systemic inflammation and enhancing cellular insulin sensitivity (37). Neutrophils are probably the dominating leukocytes in the innate arm of the immune system considering the response to damage and danger signals, which are the first leukocytes reacting to and accumulating inside the target tissues of DM (38). NLRP6 presented a positive connection with neutrophils as previously reported (39).

Based upon the DGIdb, possible compounds potentially targeting key pyroptosis genes were determined, comprising keracyanin, 9,10-phenanthrenequinone, diclofenac, phosphomethylphosphonic acid adenosyl ester, acetaminophen, cefixime, aspirin, and ustekinumab. Prior research has proposed that aspirin pretreatment mitigates inflammasome-driven pyroptosis through downregulating NF-κB/NLRP3 signaling in ischemic stroke (40). Nevertheless, experimental verification needs to be carried out for the interactions of these compounds with druggable pyroptosis molecules in T2DM.

This work determined 10 key CHMP2B-relevant genes utilizing the WGCNA along with the MCODE, namely, KNTC1, NCAPG, KIAA0101, DLGAP5, GMNN, CEP55, KIF20B, ZWILCH, MCM6, and MAD2L1. NCAPG and MAD2L1 have been demonstrated to be associated with DM and HCV-related hepatocellular carcinoma (41). DLGAP5 is connected with T2DM-attributed end-stage kidney disease among African Americans (42). Hypermethylation of KIF20B is found in the proximal tubules of diabetic kidney disease (43). Thus, prior research has unveiled the functions of key CHMP2B-relevant genes in T2DM.

Nevertheless, the limitations of this study should be pointed out. Firstly, the performance of the key pyroptosis gene-based nomogram in predicting the risk of T2DM should be validated in prospective cohorts. Secondly, the biological roles of the key pyroptosis genes in T2DM pathogenesis require to be further investigated through more experiments. Thirdly, the interactions between CHMP2B and its relevant genes should be further analyzed in T2DM.

## Conclusion

In summary, this work proposed the key pyroptosis genes in T2DM by comparing distinct machine learning approaches, composed of BAK1, CHMP2B, NLRP6, PLCG1, and TIRAP. The key pyroptosis gene-based nomogram enabled to predict the risk of T2DM for clinical application. Possible compounds that targeted the key pyroptosis genes were screened. In addition, the key CHMP2B-relevant genes were KNTC1, NCAPG, KIAA0101, DLGAP5, GMNN, CEP55, KIF20B, ZWILCH, MCM6, and MAD2L1, which might interact with CHMP2B during T2DM. Altogether, our findings offered promising molecules for the management and therapy of T2DM and its complications.

## Data availability statement

The datasets presented in this study can be found in online repositories. The names of the repository/repositories and accession number(s) can be found in the article/**Supplementary Material**.

## Author contributions

MW conceived and designed the study. HW and RW conducted most of the experiments and data analysis and wrote the manuscript. YT and QC participated in collecting the data and helped to draft the manuscript. All authors contributed to the article and approved the submitted version.

## Supplementary material

Supplementary Table 1 Landscape of the fraction of immune cell types across T2DM and control specimens.

Supplementary Table 2 The list of CHMP2B-relevant genes.

Supplementary Table 3 Selection of key CHMP2B-relevant genes *via* MCODE method.
